# Development and Validation of Quality of Life in Idiopathic Intracranial Hypertension (QOLIH) questionnaire

**DOI:** 10.3389/fneur.2026.1782362

**Published:** 2026-03-27

**Authors:** Mona Hussein, Amr Hassan, Mona A. F. Nada, Zeinab Mohammed, Rabia Gokcen Gozubatik-Celik, Banu Bayramoglu, Bilge Piri Cinar, Aynur Ozge, Amna Ibrahim, Mona Ali, Arife Çimen Atalar, Nevra Öksüz, Ahmet Hakan Bayram, Ehab Ahmed Hashish, May M. Fayez, Rehab Magdy

**Affiliations:** 1 Department of Neurology, Beni-Suef University, Beni-Suef, Egypt; 2 Department of Neurology, Cairo University, Cairo, Egypt; 3 Department of Public Health and Community Medicine, Beni-Suef University, Beni-Suef, Egypt; 4 Department of Neurology, Bakırköy Prof. Dr. Mazhar Osman Training and Research Hospital for Mental Health and Neurological Disorders, University of Health Sciences, Istanbul, Türkiye; 5 Department of Neurology, School of Medicine, Samsun University, Samsun, Türkiye; 6 Headache Clinic, Mersin, Türkiye; 7 NOROM Neuroscience and Excellence Center, Ankara, Türkiye; 8 Department of Neurology, Beni-Suef University, Beni-Suef, Egypt; 9 Department of Neurology, Istanbul Physical Therapy and Rehabilitation Training and Research Hospital, Istanbul, Türkiye; 10 Department of Neurology, Faculty of Medicine, Mersin University, Mersin, Türkiye; 11 Neuropsychiatry Department, Suez Canal University, Ismailia, Egypt; 12 Neurology Department, El Mounira General Hospital, Cairo, Egypt; 13 Department of Neurology, Cairo University, Cairo, Egypt

**Keywords:** ADL, IIH, psycho-cognitive, QOL, QOLIH

## Abstract

**Background:**

Poor quality of life (QOL) has emerged as a key morbidity in patients with idiopathic intracranial hypertension (IIH). This work aimed to develop and validate an IIH-specific quality of life assessment tool.

**Methods:**

This cross-sectional study was conducted on 146 Egyptian and 156 Turkish patients with IIH. A 14-item questionnaire was created to assess QOL in those patients; Quality of Life in IIH patients questionnaire (QOLIH). The questionnaire items were hypothesized into two domains: one domain represents activities of daily living (ADL) (Q1-7 and Q12), and the other domain represents psycho-cognitive function (Q8-11, Q13, Q14). To assess the convergent validity of this questionnaire, the following tests were also employed: the Visual Analog Scale (VAS), Headache Impact Test-6 (HIT-6), Short Form-12 Health Survey (SF-12), and Low Vision Quality-of-Life Questionnaire (LVQOL).

**Results:**

Confirmatory factor analysis (CFA) revealed that standardized factor loadings were generally acceptable (>0.60) for Q1-12. However, Q13 and Q14 consistently displayed weak loadings (<0.40), suggesting their removal. Cronbach’s Alpha for the total score of the QOLIH questionnaire (Q1–12) in the Egyptian patients was 0.940, and in the Turkish patients, it was 0.938, indicating excellent internal consistency. There were statistically significant correlations of a strong degree between QOLIH total score and HIT-6, SF-12, and LVQOL total scores in Egyptian and Turkish patients (*r* coef. > 0.7).

**Conclusion:**

The final Arabic and Turkish versions of QOLIH questionnaire consist of 12 items. Both versions are valid and reliable tools that can be used in the assessment of QOL in IIH patients.

## Introduction

Idiopathic intracranial hypertension (IIH) is a rare condition characterized by elevated intracranial pressure (ICP) with no identifiable cause. Its annual incidence rate ranges from 0.5 to 3.2 per 100,000 (1). It predominantly affects women of childbearing age with an inextricable link to obesity, as evidenced by the increasing prevalence of IIH in parallel with the global obesity epidemic (2, 3).

Headache is considered the most common symptom in 90% of IIH patients (4). It is usually bilateral, fronto-retroorbital, and pressing or explosive, occurring mainly in the morning and typically exacerbated by Valsalva maneuvers (5). Over 50% of patients with IIH were reported to have long-term sustained headaches despite the resolution of papilledema and normalization of ICP (4). Blurring of vision due to papilledema was reported to occur in approximately 50% of IIH patients, whereas complete visual loss occurs in 1–2% of patients per year (6, 7). In severe cases, patients may experience transient visual obscurations (TVOs), which are episodes of sudden, bilateral visual loss lasting a few seconds and provoked by postural changes or the Valsalva maneuver (8). The other frequently associated symptoms are double vision, pulsatile tinnitus, dizziness, ocular pain, and neck pain. It has been reported that IIH patients may also suffer from fatigue, cognitive dysfunction, anxiety, and depression (9–12). So, optimizing treatment strategies for IIH is crucial to minimize the impact of IIH on patients’ health outcomes (13).

Although poor quality of life (QOL) has emerged as a key morbidity in patients with IIH, even at diagnosis (14), there is a paucity of research evaluating QOL in IIH patients. Persistent headache is considered the main contributing factor to lower QOL in patients with IIH. Other IIH symptoms, such as visual impairment, tinnitus, fatigue, cognitive dysfunction, anxiety, and depression, may also significantly impact QOL (14). It is worth noting that QOL in IIH patients may also be affected by the wide range of side effects of carbonic anhydrase inhibitors (CAIs), the first line of treatment in IIH. These side effects were reported to occur in up to 80–100% of patients (15).

Currently, there are no IIH-specific, validated tools to assess QOL. The validated tools that are typically used in IIH clinical trials include: Short form (SF)-36 (16); Headache Impact Test (HIT-6) (17), 25-Item National Eye Institute Visual Function Questionnaire (NEI-VFQ-25) (18), Low Vision Quality-of-Life Questionnaire (LVQOL) (19) and EuroQol (20).

So, this work aimed to develop and validate an IIH-specific quality of life assessment tool to better characterize the impact of IIH symptoms on health-related quality of life.

## Methods

### Study design

This cross-sectional study represented a collaboration between two research teams from Egypt and Turkey. The initial item generation phase: literature review, patient interviews, and expert discussions, was conducted in the Egyptian cohort, and the preliminary questionnaire was developed in Arabic. Following this, the scale was translated into Turkish using forward and backward translation by two bilingual specialists. While item generation was not conducted simultaneously in both countries, the Turkish research team reviewed the translated version and confirmed its cultural appropriateness. The next steps were followed to design the Arabic QOLIH:

### Generation of quality of life in IIH patients questionnaire (QOLIH)

First, we identified items appropriate for inclusion in a disease-specific quality-of-life instrument for IIH. Three sources of items were used: (1) a list of items generated following a review of the IIH literature, (2) Patients’ views on which issues are most impactful on their QOL, (25 IIH patients were interviewed) and (3) Discussions with expert neurologists to address which issues most influence treatment decision-making.

These processes enabled the development of a preliminary 29-item questionnaire, which was subsequently condensed into a 14-item version. The reduction from the preliminary 29-item questionnaire to the final 14-item version followed a structured, multi-step approach. First, all items were evaluated during cognitive interviews with 25 patients diagnosed with IIH, who were asked to comment on item clarity, relevance, and comprehensiveness. This step allowed us to identify items perceived as ambiguous, difficult to interpret, or redundant from the patient’s perspective. Second, we conducted a systematic appraisal of the items through an updated review of the relevant literature related to IIH. This process ensured that each retained item captured a clinically meaningful symptom. Finally, the refined list of items was reviewed and discussed in detail by our multidisciplinary research team, comprising neurologists with expertise in IIH. Decisions regarding item exclusion were reached through a structured consensus process, whereby items were removed only when there was agreement that they were redundant, ambiguous, or of limited clinical relevance.

Negotiations between neurologists concluded with an agreement on two domains to which the questionnaire items are assigned: activities of daily living (ADL) restrictions and psycho-cognitive domains. The answer to each question is rated on a five-point Likert scale (never = 0, rarely = 1, sometimes = 2, very often = 3, or always = 4). The total score is the sum of all questions, with a higher score indicating poor quality of life.

Afterwards, 25 IIH patients were asked to comment on the comprehensiveness and clarity of the questions, as well as the appropriateness of the response categories, to assess cognitive validity. The questionnaire was then adjusted accordingly without further item reduction.

### Participants and procedures

Over 1 year (April 2024–April 2025), patients diagnosed with IIH, according to the modified Dandy Criteria (21), were recruited from two countries in a convenience sample. All patients were enrolled at least 12 weeks after IIH diagnosis.

Medical information, including age, body mass index (BMI), disease duration, cerebrospinal fluid (CSF) opening pressure, papilledema grading, and current daily dose of carbonic anhydrase inhibitor, were recorded.

Each patient was given a booklet containing self-report questionnaires, including the QOLIH questionnaire, as well as questionnaires in their mother tongue language (Arabic or Turkish). However, the study team members expressed their willingness to help patients with severe visual impairment.

1. Headache Impact Test-6 (HIT-6): This is a six-item scale that assesses the functions most commonly affected by headache, including daily activities, social life, psychological well-being, pain, fatigue, and attention. Each item is rated using five responses (always, very often, sometimes, never, or rarely) (22). The total HIT-6 score ranges from 36 to 78, with higher scores indicating a more severe impact (23). The validity and reliability of the Arabic and Turkish HIT-6 were confirmed by Hussein, Hassan (17) and Dikmen, Bozdağ (24), respectively.
2. Short Form-12 Health Survey (SF-12): It is a 12-item questionnaire that measures health-related quality of life. Two summary scores were obtained: the Physical Component Score (PCS) and the Mental Component Score (MCS). The former measures physical functioning, while the latter measures overall mental well-being. The two summary scores are combined to yield a total score ranging from 0 to 100. Higher scores denote a better quality of life. The Arabic version of SF-12 is valid and reliable (25), as well as the Turkish version (26).
3. Low Vision Quality-of-Life Questionnaire (LVQOL) (19): This is a 25-item scale used to assess QOL in individuals with poor vision. Each item is rated on a 5-point scale, where 1 represents “great difficulty” and 5 represents “no difficulty at all.” A higher total score, obtained by summing the individual item scores, indicates a higher QOL. The validity and reliability of LVQOL were confirmed in both of the studied languages (27, 28).

Furthermore, participants were provided with headache diaries to fill in monthly headache days (MHDs) and to assess headache severity using the visual analog scale (VAS).

### Ethics statement

Written informed consent was obtained from each participant in this study. Data were anonymous. Ethical approval was obtained from the Research Ethics Committee, Faculty of Medicine, Beni-Suef University (approval number: FMBSUREC/09022025/Hussein).

### Sample size calculation

*A priori*, a 10:1 patient-to-item ratio is considered satisfactory to obtain a sufficient sample size for the study (29). Because the number of items in this questionnaire was 14, the required sample size was determined to be at least 140 participants for each language.

### Statistical analysis

Data were analyzed using SPSS version 25 (IBM Corp., Armonk, NY, USA). The Kolmogorov–Smirnov test was used to test the normality of data. Quantitative data, including age, BMI, disease duration, CSF pressure, MHDs, VAS, HIT-6, SF-12, LVQOL, daily dose of acetazolamide, and QOLIH questionnaire, were expressed as median (interquartile range). In contrast, categorical data such as sex, grade of papilledema, and IIH symptoms were described as a number (%). Ceiling and floor effects were assessed based on the percentages of patients who achieved the best or worst total QOLIH scores. A percentage of 15% of participants was considered a threshold to detect a ceiling or a floor effect.

Confirmatory factor analysis (CFA) was used to test the Construct validity of the QOLIH questionnaire. The following fit indices were selected: root-mean-squared error of approximation (RMSEA) (30), comparative fit index (CFI) (31), chi-square, and change in chi-square given the change in degrees of freedom between models. RMSEA is a measure of the average of the residual variance and covariance; good models have RMSEA values that are at or less than 0.08 (32). CFI is an index that falls between 0 and 1, with values greater than 0.90 considered to be indicators of good-fitting models (32). Amos Version 26 was used for all analyses (33). A multi-group confirmatory factor analysis (MG-CFA) was conducted to test the cross-construct validity among Egyptian and Turkish patients. The model fit was assessed using REMSA, CFI, and chi-square. Internal consistency of the QOLIH questionnaire total score and its domains was measured using Cronbach’s alpha. Internal consistency, as measured by Cronbach’s alpha, was also reported when an item was deleted. The content validity of the QOLIH questionnaire was assessed using item domain correlations, which were analyzed using Spearman’s rho correlation coefficient. Convergent validity of the QOLIH questionnaire was also tested using Spearman’s rho correlation coefficient. A correlation coefficient of 0.00–0.1 was considered negligible correlation, 0.1–0.39 was regarded as fair correlation, 0.4–0.69 was regarded as moderate correlation, 0.7–0.89 was regarded as strong correlation, and 0.9–1 was considered very strong correlation (34). *p*-value < 0.05 was considered statistically significant. All tests were two-tailed.

## Results

### Demographics and clinical characteristics of the included Egyptian and Turkish IIH patients

This cross-sectional study was conducted on 146 Egyptian patients (12 males and 134 females) and 156 Turkish patients (47 males and 109 females) diagnosed as having IIH. The median age for the Egyptian patients was 32 (27–40) years, and for the Turkish patients, it was 34 (26.25–44) years. The median value for BMI in the Egyptian patients was 35.52 (31.09–40.17), and for the Turkish patients was 30.21 (26.69–34.52). The clinical characteristics of patients in both groups are demonstrated in Table 1.

**Table 1:** Demographics and clinical characteristics of the Egyptian and Turkish patients.

			Egyptian patients (*n* = 146)	Turkish patients (*n* = 156)
Age [median (IQR)]			32 (27–40)	34 (26.25–44)
Sex	Male [*n* (%)]		12 (8.2%)	47 (30.1%)
	Females [*n* (%)]		134 (91.8%)	109 (69.9%)
BMI [median (IQR)]			35.52 (31.09–40.17)	30.21 (26.69–34.52)
Disease duration in years [median (IQR)]			2 (0.5–3)	0.75 (0.33–2)
Grade of papilledema [*n* (%)]	Rt eye	No papilledema	4 (2.7%)	0
		Grade I	41 (28.1%)	47 (30.1%)
		Grade II	62 (42.5%)	52 (33.3%)
		Grade III	32 (21.9%)	41 (26.3%)
		Grade IV	7 (4.8%)	15 (9.6%)
		Grade V	0	1 (0.6)
	Lt eye	No papilledema	4 (2.7%)	0
		Grade I	39 (26.7%)	41 (26.3%)
		Grade II	65 (44.5%)	57 (36.5%)
		Grade III	28 (19.2%)	40 (25.6%)
		Grade IV	8 (5.5%)	14 (9.0%)
		Grade V	2 (1.4%)	4 (2.6%)
CSF pressure [median (IQR)]			280 (260–340)	300 (280–360)
MHDs [median (IQR)]			15 (8–30)	13 (5–25)
VAS [median (IQR)]			7 (5–8)	7 (5–8)
HIT-6 total score [median (IQR)]			60 (53–67)	63 (54.5–66.5)
SF-12 [median (IQR)]	PCS		35.69 (29.6200–42.8475)	34.05 (28.21–42.49)
	MCS		34.6 (28.26–41.38)	34.56 (27.69–40.42)
	Total score		68.1 (60.71–81.74)	66.12 (58.29–81.43)
LVQOL [median (IQR)]			79 (62–94)	70 (59–95)
IIH symptoms [*n* (%)]	Blurring of vision		128 (87.7%)	93 (59.6%)
	Diplopia		57 (39.0%)	55 (35.3&)
	TVOs		61 (41.8%)	72 (46.2%)
	Tinnitus		97 (66.4%)	78 (50.0%)
	Dizziness		84 (57.5%)	77 (49.4%)
	Ocular pain		65 (44.5%)	40 (25.6%)
Daily dose of acetazolamide [median (IQR)]			1,000 (750–1,500)	1,000 (750–1,250)

BMI, Body mass index; CSF, Cerebrospinal fluid; HIT-6, Headache Impact Test-6; IIH, Idiopathic intracranial hypertension; LVQOL, Low vision quality of life; MCS, Mental component score; MHDs, Monthly headache days; PCS, Physical component score; SF-12, 12-item Short Form; TVOs, Transient visual obscurations; VAS, Visual analog scale.

### Construct validity analysis of the QOLIH questionnaire in Egyptian and Turkish IIH patients using CFA

**Figure 1:**
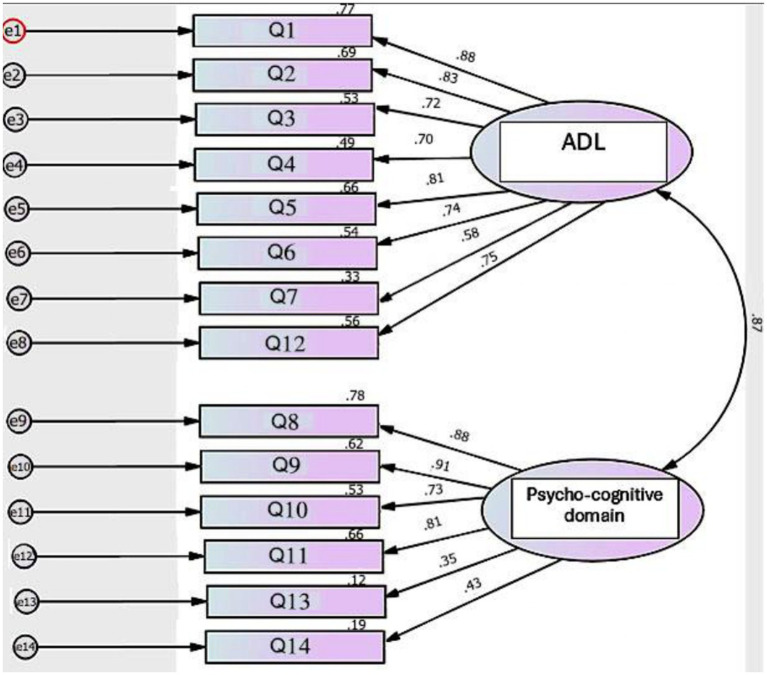
CFA of QOLIH questionnaire in Egyptian patients. ADL, Activities of daily living, CFA, confirmatory factor analysis, QOLIH questionnaire, quality of life in IIH patients questionnaire.

**Figure 2:**
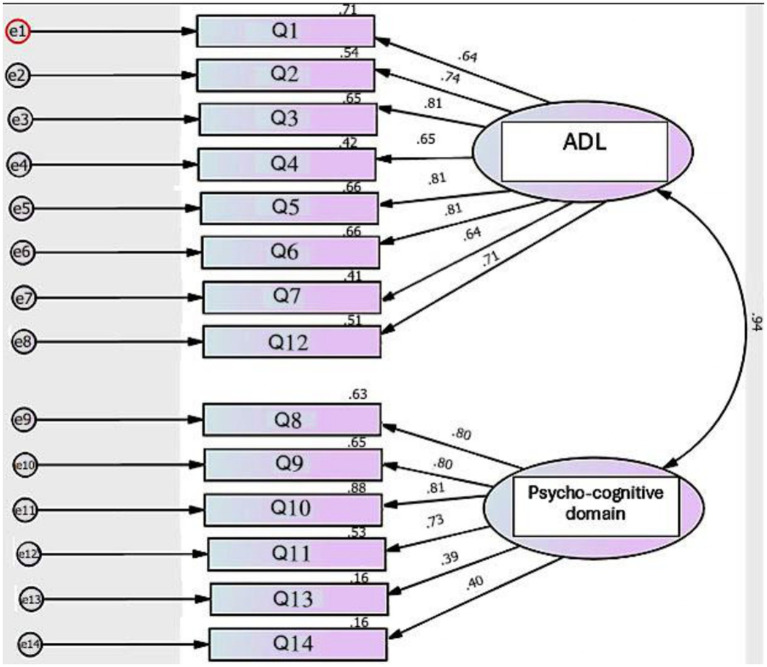
CFA of QOLIH questionnaire in Turkish patients. ADL, Activities of daily living, CFA, confirmatory factor analysis, QOLIH questionnaire, Quality of life in IIH patients questionnaire.

Two domains were created: one domain represents ADL (Q1–7 and Q12), and the other domain represents psycho-cognitive function (Q8–11 and Q13–14). In the Egyptian patients, model fit was acceptable: *χ*^2^(76) = 196.37, *p* < 0.001, CFI = 0.909, TLI = 0.891, RMSEA = 0.105. The Turkish version showed weaker model fit: *χ*^2^(76) = 241.11, *p* < 0.001, CFI = 0.879, TLI = 0.855, RMSEA = 0.118. In both Egyptian and Turkish patients, standardized factor loadings were generally acceptable (greater than 0.60) for Q1–12. However, Q13 (In the last month, did you feel like you are a burden to others because of your illness?) and Q14 (In the last month, did you have any suicidal thoughts because of your illness?) consistently displayed weak loadings (<0.40) and low squared multiple correlations (*R*^2^ < 0.20), indicating poor performance (Figures 1, 2).

MG-CFA was conducted to test the cross-construct validity of the QOLIH questionnaire among Egyptian and Turkish patients after removing Q13 and Q14, which displayed low loading in the preliminary CFA (Table 2). The CMIN/df ratio of 2.84 falls within the acceptable range (1–3), supporting an adequate model fit. CFI was 0.923, indicating that the model fits the data well compared to a null model. RMSEA was 0.078, suggesting an acceptable fit.

**Table 2:** Standardized factor loadings of QOLIH items in Egyptian and Turkish patients.

Item	Domains	Standardized loading in Egyptian patients	Standardized loading in Turkish patients
Q1	ADL domain	0.876	0.841
Q2	ADL domain	0.833	0.735
Q3	ADL domain	0.724	0.807
Q4	ADL domain	0.699	0.642
Q5	ADL domain	0.812	0.815
Q6	ADL domain	0.734	0.812
Q7	ADL domain	0.578	0.635
Q12	ADL domain	0.751	0.715
Q8	Psycho-cognitive domain	0.880	0.809
Q9	Psycho-cognitive domain	0.894	0.812
Q10	Psycho-cognitive domain	0.734	0.817
Q11	Psycho-cognitive domain	0.821	0.723

ADL, Activities of daily living; CFA, Confirmatory factor analysis; QOLIH questionnaire, Quality of life in IIH patients questionnaire.

### Scores of QOLIH questionnaire and its domains in Egyptian and Turkish IIH patients

Based on the results of CFA, Q13 and Q14 were removed. The median total score of the QOLIH questionnaire (Q1–12) in Egyptian patients was 21 (15–31.25), and in Turkish patients, it was 27.5 (16–33) (Table 3). No ceiling or floor effects were detected in the total score of the QOLIH questionnaire in Egyptian patients, as only one participant (0.68%) achieved the lowest total score (3), and one participant (0.68%) achieved the highest total score (46). Additionally, no ceiling or floor effects were detected in the total score of the QOLIH questionnaire among Turkish patients, as only one participant (0.64%) achieved the lowest total score (1), and two participants (1.28%) achieved the highest total score (46).

**Table 3:** Scores of QOLIH questionnaire and its domains in Egyptian and Turkish IIH patients.

QOLIH questionnaire	Egyptian patients (*n* = 146) [median (IQR)]	Turkish patients (*n* = 156) [median (IQR)]
ADL domain		
1. In the last month, did you have difficulties in doing your work or household tasks because of your headache and/or blurred vision?	2 (2–3)	2 (2–3)
2. In the last month, did you have difficulties in watching electronic devices (TV, computer, tablet or cell phone) because of your headache and/or blurred vision?	2 (1–3)	2 (1–3)
3. In the last month, did you have difficulties in reading ordinary print because of your blurred vision?	2 (1–3)	2 (1–3)
4. In the last month, did you have any sort of ocular pain or discomfort?	1 (1–3)	2 (1–3)
5. In the last month, was your performance level at work reduced because of your headache and/or blurred vision?	2 (1–3)	2 (1–3)
6. In the last month, were your social activities affected because of your headache and/or blurred vision?	2 (1–2)	2 (1–3)
7. In the last month, how much did the side effects of your medications such as parasthesia annoy you?	0 (0–2)	2 (0–3)
12. In the last month, did you feel you did not have enough energy to do simple tasks?	2 (1–3)	2 (1–3)
ADL domain score	15 (11–21)	18 (11–23)
Psycho-cognitive domain		
8. In the last month, did you become irritable because of your headache and/or blurred vision?	2 (1–3)	2 (1–3)
9. In the last month, did you feel sadness and frustration because of your headache and/or blurred vision?	2 (1–2.25)	2 (1–3)
10. In the last month, Did you have non-refreshing sleep because of your headache?	1 (0–2)	2 (1–3)
11. In the last month, did you have difficulties in concentrating at work or daily activities because of your headache and/or blurred vision?	2 (1–3)	2 (1–3)
Psycho-cognitive domain score	6 (4–11)	8.5 (4.25–11)
Total score of QOLIH questionnaire	21 (15–31.25)	27.5 (16–33)

ADL, Activities of daily living; QOLIH questionnaire, Quality of life in IIH patients questionnaire.

### Reliability estimates and content validity of QOLIH questionnaire scores in Egyptian and Turkish IIH patients

Cronbach’s Alpha for the total score of the QOLIH questionnaire (Q1–12) in the Egyptian patients was 0.940, and in the Turkish patients, it was 0.938, indicating excellent internal consistency (Table 4). The correlation coefficients between most items of the QOLIH questionnaire and their corresponding domains were significantly higher than those of the other domains or the total QOLIH score in both Egyptian and Turkish patients (Table 5).

**Table 4:** Internal consistency of QOLIH questionnaire and its domains in Egyptian and Turkish IIH patients.

QOLIH questionnaire		Egyptian patients (*n* = 146)	Turkish patients (*n* = 156)
Cronbach’s alpha	ADL domain score	0.907	0.909
	Psycho-cognitive domain score	0.899	0.865
	Total QOLIH score	0.940	0.938
Cronbach’s alpha if item deleted	Q1	0.933	0.931
	Q2	0.934	0.934
	Q3	0.938	0.931
	Q4	0.937	0.937
	Q5	0.934	0.931
	Q6	0.936	0.931
	Q7	0.941	0.937
	Q8	0.932	0.933
	Q9	0.932	0.933
	Q10	0.938	0.932
	Q11	0.933	0.933
	Q12	0.934	0.935

ADL, Activities of daily living; QOLIH questionnaire, Quality of life in IIH patients questionnaire.

**Table 5:** Items domains correlations of QOLIH questionnaire in Egyptian and Turkish patients.

	Egyptian patients (*n* = 146)						Turkish patients (*n* = 156)					
	ADL domain score		Psycho-cognitive domain score		Total QOLIH score		ADL domain score		Psycho-cognitive domain score		Total QOLIH score	
	(*r*) coef.	*p*-value	(*r*) coef.	*p*-value	(*r*) coef.	*p*-value	(*r*) coef.	*p*-value	(*r*) coef.	*p*-value	(*r*) coef.	*p*-value
ADL domain												
Q1	0.862	<0.001*	0.714	<0.001*	0.835	<0.001*	0.831	<0.001*	0.692	<0.001*	0.812	<0.001*
Q2	0.814	<0.001*	0.648	<0.001*	0.777	<0.001*	0.787	<0.001*	0.638	<0.001*	0.760	<0.001*
Q3	0.745	<0.001*	0.566	<0.001*	0.704	<0.001*	0.820	<0.001*	0.718	<0.001*	0.824	<0.001*
Q4	0.727	<0.001*	0.596	<0.001*	0.699	<0.001*	0.722	<0.001*	0.549	<0.001*	0.685	<0.001*
Q5	0.831	<0.001*	0.669	<0.001*	0.793	<0.001*	0.805	<0.001*	0.702	<0.001*	0.798	<0.001*
Q6	0.784	<0.001*	0.644	<0.001*	0.761	<0.001*	0.813	<0.001*	0.702	<0.001*	0.799	<0.001*
Q7	0.513	<0.001*	0.523	<0.001*	0.539	<0.001*	0.714	<0.001*	0.542	<0.001*	0.681	<0.001*
Q12	0.772	<0.001*	0.727	<0.001*	0.786	<0.001*	0.698	<0.001*	0.618	<0.001*	0.687	<0.001*
Psycho-cognitive domain												
Q8	0.756	<0.001*	0.896	<0.001*	0.840	<0.001*	0.643	<0.001*	0.868	<0.001*	0.758	<0.001*
Q9	0.761	<0.001*	0.906	<0.001*	0.855	<0.001*	0.681	<0.001*	0.862	<0.001*	0.782	<0.001*
Q10	0.632	<0.001*	0.826	<0.001*	0.737	<0.001*	0.737	<0.001*	0.854	<0.001*	0.813	<0.001*
Q11	0.759	<0.001*	0.859	<0.001*	0.832	<0.001*	0.712	<0.001*	0.761	<0.001*	0.759	<0.001*

ADL, Activities of daily living; QOLIH questionnaire, Quality of life in IIH patients questionnaire.

(*r*): Spearman correlation, *p-value <0.05 is considered significant.

### Convergent validity analysis of QOLIH questionnaire in Egyptian and Turkish IIH patients

Regarding Egyptian patients, there were statistically significant correlations of a very strong degree between QOLIH questionnaire total score and LVQOL total score (*r* = −0.917, *p*-value < 0.001), of strong degree between QOLIH questionnaire total score and both HIT-6 total score (*r* = 0.855, *p*-value < 0.001) and SF-12 total score (*r* = −0.795, *p*-value < 0.001), of moderately strong degree between QOLIH questionnaire total score and both MHDs (*r* = 0.637, *p*-value < 0.001) and VAS (*r* = 0.654, *p*-value < 0.001), of fair degree between QOLIH questionnaire total score and the daily dose of acetazolamide (*r* = 0.407, *p*-value < 0.001), and of poor degree between QOLIH questionnaire total score and BMI (*r* = 0.193, *p*-value = 0.020) (Table 6).

**Table 6:** Convergent validity analysis of total score of QOLIH in Egyptian and Turkish patients.

		QOLIH questionnaire total score in Egyptian patients		QOLIH questionnaire total score in Turkish patients	
		(*r*) coef.	*p*-value	(*r*) coef.	*p*-value
BMI		0.193	0.020*	−0.054	0.502
Disease duration in years		0.087	0.295	0.020	0.808
CSF pressure		0.139	0.094	0.050	0.532
MHDs		0.637	<0.001*	0.514	<0.001*
VAS		0.654	<0.001*	0.462	<0.001*
HIT-6 total score		0.855	<0.001*	0.712	<0.001*
SF-12	PCS	−0.621	<0.001*	−0.643	< 0.001*
	MCS	−0.613	<0.001*	−0.614	< 0.001*
	Total score	−0.795	<0.001*	−0.831	< 0.001*
LVQOL		−0.917	<0.001*	−0.881	<0.001*
Daily dose of acetazolamide		0.407	<0.001*	0.437	<0.001*

BMI, Body mass index; CSF, Cerebrospinal fluid; HIT-6, Headache Impact Test-6; LVQOL, Low vision quality of life; MCS, Mental component score; MHDs, Monthly headache days; PCS, Physical component score; SF-12, 12-item Short Form; VAS, Visual analog scale.

(*r*) Coef: Spearman’s correlation coefficient, *Correlation is significant at the <0.05 level (2-tailed).

Regarding Turkish patients, there were statistically significant correlations of a strong degree between QOLIH questionnaire total score and HIT-6 total score (*r* = 0.712, *p*-value < 0.001), SF-12 total score (*r* = −0.831, *p*-value < 0.001), and LVQOL total score (*r* = −0.881, *p*-value < 0.001), of moderately strong degree between QOLIH questionnaire total score and MHDs (*r* = 0.514, *p*-value < 0.001), and of fair degree between QOLIH questionnaire total score and both VAS (*r* = 0.462, *p*-value < 0.001) and the daily dose of acetazolamide (*r* = 0.437, *p*-value < 0.001) (Table 6).

## Discussion

A QOL assessment tool is necessary for patients with IIH in clinical practice to gain a deeper understanding of patients’ needs and goals, as well as to determine whether management is being effectively met.

The QOLIH covers the entire range of QOL issues in patients with IIH. In addition to measuring headaches and visual-related quality of life, it also tracks other symptoms that some physicians may overlook, such as cognitive impairment, sleep disturbance, and psychological well-being. Indeed, previous studies evaluating the QOL in IIH patients have relied solely on assessing headache and visual impairment, without paying attention to other symptoms (14, 35, 36), although about half of the IIH patients acknowledge that sleep disturbances, depression, and cognitive impairment are major contributors to their symptom burden (37, 38). This provided a basis for assuming ADL and psycho-cognitive function domains. Targeting all-encompassing information can help identify cases where further management may be necessary to improve an individual’s quality of life.

The questionnaire is also of an acceptable length (14 items) and takes about 5–8 min to complete, which enhances its use in clinical and many research settings. On the other hand, it does not seem to put undue stress on anybody with poor vision.

The present study included two samples of two distinct geographic regions. However, the two language versions of QOLIH could meet the classic psychometric quality criteria of validity and reliability. Nonetheless, both items 13 and 14 displayed low factor loadings in the CFA (<0.40), as demonstrated by the two language versions, and were therefore excluded from the analysis.

In terms of convergent validity, the QOLIH total scores demonstrated a strong correlation with those of other widely used quality of life QoL scales among IIH patients, including the HIT-6, LVQOL, and SF-12 (*r* > 0.7). On the other hand, the correlations were moderate in magnitude with MHDs, in the range of *r* = 0.5–0.6. These findings align with earlier literature, which showed that headache severity has more robust correlates with QOL measures than headache frequency (39, 40). Therefore, headache severity should be regarded as the most important outcome measure in clinical trial settings for IIH, as it has the most significant impact on QOL. In contrast, a poor correlation was noted between the Egyptian QOLIH-total score and BMI (*r* = 0.193), while no correlation was observed with the Turkish version. Likewise, Digre, Bruce (14) found that obesity alone cannot explain the poor QOL in IIH patients.

Furthermore, the internal consistency of the QOLIH, as evidenced by Cronbach’s alpha greater than 0.9, indicated that each of the two language versions possesses properties of a reliable instrument. Item-domain correlations revealed adequate content validity of the test.

Finally, it is worthy of discussion that due to the lack of objective biomarkers in headache practice, Patient-Reported Outcome Measures (PROMs) are essential in evaluating treatment responses, supporting clinical decision-making and better-designed studies. However, they cannot grasp the full experience of living with headache disorders, as other elements relating to the disability of headache disorders are neglected, including reduced work productivity, economic impacts, and the interictal burden (41).

Future studies adopting the English validation of QOLIH, as well as validations in other languages, are awaited. The main limitation of the present study is its cross-sectional design, which hinders the examination of the questionnaire’s longitudinal properties, such as sensitivity to change and predictive validity. Further studies are required to investigate the ability of the QOLIH to detect changes throughout the disease course, patient response to treatment, and its role in predicting prognosis. A further major limitation is the absence of test–retest reliability assessment. Subsequent studies are therefore needed to administer the QOLIH at two time points in clinically stable patients (e.g., 2-week interval) to formally evaluate test–retest reliability and confirm the stability of QOLIH scores over time. Another limitation is that we proceeded directly to CFA based on a hypothesized structure derived from expert consensus, without conducting an EFA on a separate subsample. Although the overall sample size was adequate for CFA, it did not permit splitting the data to perform both EFA and CFA. Future studies with larger cohorts should incorporate both EFA and CFA to provide a more rigorous and comprehensive psychometric validation of the QOLIH.

## Conclusion

The QOLIH questionnaire is a novel disease-specific measure to assess the health-related quality of life in patients with IIH. The current findings suggest that the instrument possesses satisfactory psychometric properties, including validity and reliability, which support its use in daily medical practice and clinical research.

## Data Availability

The raw data supporting the conclusions of this article will be made available by the authors without undue reservation.
