# Supplementary material for: Development and Validation of Quality of Life in Idiopathic Intracranial Hypertension (QOLIH) questionnaire
Source: Front Neurol. 2026 Mar 27;17:1782362. doi: 10.3389/fneur.2026.1782362 (PMC13065696; doi:10.3389/fneur.2026.1782362)
Supplement: Supplementary file 1 [file Table_1.docx]

**Idiopathic Intracranial Hypertension Quality of Life Questionnaire (IIH-QOL)**

1. In the last month, did you have difficulties in doing your work or household tasks because of your headache and/or blurred vision?

| a. Never | b. Rarely | 1. Sometimes | 1. Often | 1. Always |
| --- | --- | --- | --- | --- |

1. In the last month, did you have difficulties in watching electronic devices (TV, computer, tablet or cell phone) because of your headache and/or blurred vision?

| a. Never | b. Rarely | 1. Sometimes | 1. Often | 1. Always |
| --- | --- | --- | --- | --- |

1. In the last month, did you have difficulties in reading ordinary print because of your blurred vision?

| a. Never | b. Rarely | 1. Sometimes | 1. Often | 1. Always |
| --- | --- | --- | --- | --- |

1. In the last month, did you have any sort of ocular pain or discomfort?

| a. Never | b. Rarely | 1. Sometimes | 1. Often | 1. Always |
| --- | --- | --- | --- | --- |

1. In the last month, was your performance level at work reduced because of your headache and/or blurred vision?

| a. Never | b. Rarely | 1. Sometimes | 1. Often | 1. Always |
| --- | --- | --- | --- | --- |

1. In the last month, were your social activities affected because of your headache and/or blurred vision?

| a. Never | b. Rarely | 1. Sometimes | 1. Often | 1. Always |
| --- | --- | --- | --- | --- |

1. In the last month, how much did the side effects of your medications such as parasthesia annoy you?

| a. Never | b. Rarely | 1. Sometimes | 1. Often | 1. Always |
| --- | --- | --- | --- | --- |

1. In the last month, did you become irritable because of your headache and/or blurred vision?

| a. Never | b. Rarely | 1. Sometimes | 1. Often | 1. Always |
| --- | --- | --- | --- | --- |

1. In the last month, did you feel sadness and frustration because of your headache and/or blurred vision?

| a. Never | b. Rarely | 1. Sometimes | 1. Often | 1. Always |
| --- | --- | --- | --- | --- |

1. In the last month, did you have non-refreshing sleep because of your headache?

| a. Never | b. Rarely | 1. Sometimes | 1. Often | 1. Always |
| --- | --- | --- | --- | --- |

1. In the last month, did you have difficulties in concentrating at work or daily activities because of your headache and/or blurred vision?

| a. Never | b. Rarely | 1. Sometimes | 1. Often | 1. Always |
| --- | --- | --- | --- | --- |

1. In the last month, did you feel you didn’t have enough energy to do simple tasks?

| a. Never | b. Rarely | 1. Sometimes | 1. Often | 1. Always |
| --- | --- | --- | --- | --- |
