# Supplementary material for: Development and Validation of Quality of Life in Idiopathic Intracranial Hypertension (QOLIH) questionnaire
Source: Front Neurol. 2026 Mar 27;17:1782362. doi: 10.3389/fneur.2026.1782362 (PMC13065696; doi:10.3389/fneur.2026.1782362)
Supplement: Supplementary file 2 [file Table_2.docx]

İdiyopatik İntrakranial hipertansiyonda yaşam kalitesi anketi (QOLIH)

1. Son bir ayda, baş ağrınız ve/veya bulanık görmeniz nedeniyle işinizi veya ev işlerinizi yaparken zorluk yaşadınız mı?
2. Hiç b. Nadiren c. Bazen d. Sıklıkla e. Her zaman
3. Son bir ayda baş ağrınız ve/veya bulanık görmeniz nedeniyle elektronik cihazları (TV, bilgisayar, tablet veya cep telefonu) izlemekte zorluk çektiniz mi?
4. Hiç b. Nadiren c. Bazen d. Sıklıkla e. Her zaman
5. Son bir ayda bulanık görmeniz nedeniyle normal yazıları okumakta zorluk çektiniz mi?

a. Hiç b. Nadiren c. Bazen d. Sıklıkla e. Her zaman

1. Son bir ayda gözünüzde herhangi bir ağrı veya rahatsızlık hissettiniz mi?

a. Hiç b. Nadiren c. Bazen d. Sıklıkla e. Her zaman

1. Son bir ayda baş ağrınız ve/veya bulanık görmeniz nedeniyle iş yerinizdeki performansınızda düşüş oldu mu?

a. Hiç b. Nadiren c. Bazen d. Sıklıkla e. Her zaman

1. Son bir ayda baş ağrınız ve/veya bulanık görmeniz nedeniyle sosyal aktiviteleriniz etkilendi mi?

a. Hiç b. Nadiren c. Bazen d. Sıklıkla e. Her zaman

1. Son bir ayda kullandığınız ilaçların yan etkileri sizi ne kadar rahatsız etti?

a. Hiç b. Nadiren c. Bazen d. Sıklıkla e. Her zaman

1. Son bir ayda baş ağrınız ve/veya bulanık görmeniz nedeniyle sinirli hissetiniz mi?

a. Hiç b. Nadiren c. Bazen d. Sıklıkla e. Her zaman

1. Son bir ayda baş ağrınız ve/veya bulanık görmeniz nedeniyle üzüntü ve hayal kırıklığı hissettiniz mi?

a. Hiç b. Nadiren c. Bazen d. Sıklıkla e. Her zaman

1. Son bir ayda baş ağrınız dinlendirici olmayan uykuya neden oldu mu?

a. Hiç b. Nadiren c. Bazen d. Sıklıkla e. Her zaman

1. Son bir ayda baş ağrınız ve/veya bulanık görmeniz nedeniyle işinizde veya günlük aktivitelerinizde konsantre olmada zorluk yaşadınız mı?

a. Hiç b. Nadiren c. Bazen d. Sıklıkla e. Her zaman

1. Son bir ayda, basit işleri yapacak enerjinizin olmadığını hissettiniz mi?

a. Hiç b. Nadiren c. Bazen d. Sıklıkla e. Her zaman

1. Son bir ayda hastalığınız nedeniyle başkalarına yük olduğunuzu hissettiniz mi?

a. Hiç b. Nadiren c. Bazen d. Sıklıkla e. Her zaman

1. Son bir ayda hastalığınız nedeniyle intihar düşünceleriniz oldu mu?

a. Hiç b. Nadiren c. Bazen d. Sıklıkla e. Her zaman
